# Supplementary material for: Dynomics: A Novel and Promising Approach for Improved Breast Cancer Prognosis Prediction
Source: J Pers Med. 2023 Jun 15;13(6):1004. doi: 10.3390/jpm13061004 (PMC10303631; doi:10.3390/jpm13061004)
Supplement: Supplementary file 1 [file jpm-13-01004-s001.zip › jpm-2418638-supplementary.pdf]

Supplementary Materials

Table S1. List of the extracted radiomic features. Radiomic features were extracted within the lesion and reference tissue volumes of interest using Python software and the Pyradiomics module. Features included first order, shape (2D), shape (3D), gray level cooccurrence matrix (GLCM), gray level size zone matrix (GLSZM), gray level run length matrix (GLRLM), neighboring gray tone difference matrix (NGTDM), and gray level dependence matrix (GLDM).

**Feature Name**	**Feature Code**
Shape Elongation	1
Shape Flatness	2
Shape Least Axis Length	3
Shape Major Axis Length	4
Shape Maximum 2D Diameter Column	5
Shape Maximum 2D Diameter Row	6
Shape Maximum 2D Diameter Slice	7
Shape Maximum 3D Diameter	8
Shape Mesh Volume	9
Shape Minor Axis Length	10
Shape Sphericity	11
Shape Surface Area	12
Shape Surface Volume Ratio	13
Shape Voxel Volume	14
First Order 10 Percentile	15
First Order 90 Percentile	16
First Order Energy	17
First Order Entropy	18
First Order Interquartile Range	19
First Order Kurtosis	20
First Order Maximum	21
First Order Mean Absolute Deviation	22
First Order Mean	23
First Order Median	24
First Order Minimum	25
First Order Range	26
First Order Robust Mean Absolute Deviation	27
First Order Root Mean Squared	28
First Order Skewness	29
First Order Total Energy	30
First Order Uniformity	31
First Order Variance	32
GLCM Autocorrelation	33
GLCM Cluster Prominence	34
GLCM Cluster Shade	35
GLCM Cluster Tendency	36
GLCM Contrast	37
GLCM Correlation	38
GLCM Difference Average	39

GLCM Difference Entropy	40
GLCM Difference Variance	41
GLCM Id	42
GLCM Idm	43
GLCM Idmn	44
GLCM Idn	45
GLCM Imc1	46
GLCM Imc2	47
GLCM Inverse Variance	48
GLCM Joint Average	49
GLCM Joint Energy	50
GLCM Joint Entropy	51
GLCM MCC	52
GLCM Maximum Probability	53
GLCM Sum Average	54
GLCM Sum Entropy	55
GLCM Sum Squares	56
GLDM Dependence Entropy	57
GLDM Dependence Non-Uniformity	58
GLDM Dependence Non-Uniformity Normalized	59
GLDM Dependence Variance	60
GLDM Gray Level Non-Uniformity	61
GLDM Gray Level Variance	62
GLDM High Gray Level Emphasis	63
GLDM Large Dependence Emphasis	64
GLDM Large Dependence High Gray Level Emphasis	65
GLDM Large Dependence Low Gray Level Emphasis	66
GLDM Low Gray Level Emphasis	67
GLDM Small Dependence Emphasis	68
GLDM Small Dependence High Gray Level Emphasis	69
GLDM Small Dependence Low Gray Level Emphasis	70
GLRLM Gray Level Non-Uniformity	71
GLRLM Gray Level Non-Uniformity Normalized	72
GLRLM Gray Level Variance	73
GLRLM High Gray Level Run Emphasis	74
GLRLM Long Run Emphasis	75
GLRLM Long Run High Gray Level Emphasis	76
GLRLM Long Run Low Gray Level Emphasis	77
GLRLM Low Gray Level Run Emphasis	78
GLRLM Run Entropy	79
GLRLM Run Length Non-Uniformity	80
GLRLM Run Length Non-Uniformity Normalized	81
GLRLM Run Percentage	82
GLRLM Run Variance	83
GLRLM Short Run Emphasis	84
GLRLM Short Run High Gray Level Emphasis	85
GLRLM Short Run Low Gray Level Emphasis	86

GLSZM Gray Level Non-Uniformity	87
GLSZM Gray Level Non-Uniformity Normalized	88
GLSZM Gray Level Variance	89
GLSZM High Gray Level Zone Emphasis	90
GLSZM Large Area Emphasis	91
GLSZM Large Area High Gray Level Emphasis	92
GLSZM Large Area Low Gray Level Emphasis	93
GLSZM Low Gray Level Zone Emphasis	94
GLSZM Size Zone Non-Uniformity	95
GLSZM Size Zone Non-Uniformity Normalized	96
GLSZM Small Area Emphasis	97
GLSZM Small Area High Gray Level Emphasis	98
GLSZM Small Area Low Gray Level Emphasis	99
GLSZM Zone Entropy	100
GLSZM Zone Percentage	101
GLSZM Zone Variance	102
NGTDM Busyness	103
NGTDM Coarseness	104
NGTDM Complexity	105
NGTDM Contrast	106
NGTDM Strength	107
